# Tests of Death

**Published:** 1873-04

**Authors:** 


### TESTS OF DEATH.

In every case of death the blood ceases to flow and we cease to breathe. Sometimes there is so little breath that a polished mirror is not tarnished, and yet there is life.

A polished needle stuck into the flesh is moistened if there is life ; if there is none, the needle is as bright as before.

A burning candle or hot iron to the skin raises a blister if there is life, otherwise none.

When life is not entirely extinct, animation has been restored by cutting the finger so as to cause it to bleed, thus setting the circulation in motion again.

The latest suggestion is to tie a cord tightly around the finger or toe; if there is the very slightest circulation remaining, the skin between the cord and the extremity begins to distend, blacken, or redden, if there is life, and the whole space shows a uniform bluish red, except that there is a ring of white at the ligature.

The very last anxiety of the great Washington was that he should not be buried alive, and the very last words he ever uttered, "It is well," were in response to the attendant that he understood Washington's wishes in that respect.

